# MR Imaging-Histology Correlation by Tailored 3D-Printed Slicer in Oncological Assessment

**DOI:** 10.1155/2019/1071453

**Published:** 2019-05-29

**Authors:** D. Baldi, M. Aiello, A. Duggento, M. Salvatore, C. Cavaliere

**Affiliations:** ^1^IRCCS SDN, Napoli, Italy; ^2^Department of Biomedicine and Prevention, University of Rome “Tor Vergata”, Rome, Italy

## Abstract

3D printing and reverse engineering are innovative technologies that are revolutionizing scientific research in the health sciences and related clinical practice. Such technologies are able to improve the development of various custom-made medical devices while also lowering design and production costs. Recent advances allow the printing of particularly complex prototypes whose geometry is drawn from precise computer models designed on in vivo imaging data. This review summarizes a new method for histological sample processing (applicable to e.g., the brain, prostate, liver, and renal mass) which employs a personalized mold developed from diagnostic images through computer-aided design software and 3D printing. Through positioning the custom mold in a coherent manner with respect to the organ of interest (as delineated by in vivo imaging data), the cutting instrument can be precisely guided in order to obtain blocks of tissue which correspond with high accuracy to the slices imaged. This approach appeared crucial for validation of new quantitative imaging tools, for an accurate imaging-histopathological correlation and for the assessment of radiogenomic features extracted from oncological lesions. The aim of this review is to define and describe 3D printing technologies which are applicable to oncological assessment and slicer design, highlighting the radiological and pathological perspective as well as recent applications of this approach for the histological validation of and correlation with MR images.

## 1. Introduction

The need to better understand cancer pathogenesis for diagnostic and prognostic purposes has boosted the development of different imaging techniques (such as positron emission tomography (PET), single-photon emission computed tomography (SPECT), computed tomography (CT), and magnetic resonance imaging (MRI)) which are able to characterize anatomical, functional, and molecular features of oncological lesions in a noninvasive and quantitative way [1, 2]. Among these, MRI is a multiparametric modality which can simultaneously provide morphological as well functional contrasts, hence affording the largest possible share of information detectable by a single technique. In turn, this allows the simultaneous detection of different processes concurring to the carcinogenesis pathway [3]. For example, multiparametric MRI has facilitated significant advances in prostate cancer imaging. Specifically, modern MR examinations in prostate cancer are able to take advantage of high spatial resolution (<1 mm) and of the combination of functional as well as morphological images, hence integrating information on cellularity and vascularization of possible lesions [4].

Nevertheless, MR assessment of oncological lesions is affected by several drawbacks that limit its ability in differential diagnosis, diagnostic accuracy, and predictive power for diagnosis, as well as prediction of treatment response [5, 6]. These limitations are mainly due to the lack of matched histological data which, in spite of its invasiveness, still represents the gold standard for lesion assessment and characterization. Four major issues can introduce bias when studying correlations between imaging and histological data [7]. First, differences in orientation between the imaging scan planes and the surgical sample can determine a significant mismatch. Second, the tissue deformation which occurs when the histological sample is placed outside its anatomical background (due to the lack of tension and mechanical compression provided by supporting tissues and vascularization) determines important and locally nonlinear alignment inconsistencies. Third, the different spatial resolution of the two methods (1–5 mm compared to 3–5 *μ*m, for MR and histology respectively) does not allow accurate superposition. Finally, differences in contrast resolution increase the difficulty in assessing the accuracy of any coregistration processes.

While several groups have developed standard devices to support the tissue slicing commonly performed manually by pathologist, difficulties related to spatial sample orientation and its deformation during the cutting phase remained unresolved [8]. In this context, the introduction of custom-made slicers has also been fueled by the recent employment of the 3D printers for biomedical purposes. This procedure is often described as a reverse engineering of diagnostic images for constructing complex but anatomically constrained and accurate prototypes [9]. The applications of this approach include the surgical planning and training, patient education for improved compliance, dedicated phantom prototyping, and tailored prostheses design. The latter application is made possible by technologies like stereolithography (SLA) and digital light processing (DLP), which have become more affordable and less expensive and employ photopolymers with a high degree of biocompatibility [10–12]. Also, unlike standard slicer models [13, 14], the customized molds allow to execute the histological assessment following the same orientation and slicing of the MRI scans through a standardized procedural workflow.

The aim of this review is to define and describe 3D printing technologies which are applicable to oncological assessment and slicer design, highlighting the radiological and pathological perspective as well as recent applications of this approach for the histological validation of and correlation with MR images.

The technology of additive manufacturing and the different existing 3D printing technologies will be initially described. It will be emphasized that the integration between pathology and radiology data can improve the clinical routine, particularly in oncology.

The applications of slicer in the oncological literature will be described for various anatomical districts such as the prostate, brain (in clinical and preclinical settings), liver, and kidney.

## 2. Reverse Engineering Workflow and 3D Printing Technology

**Figure 1:**
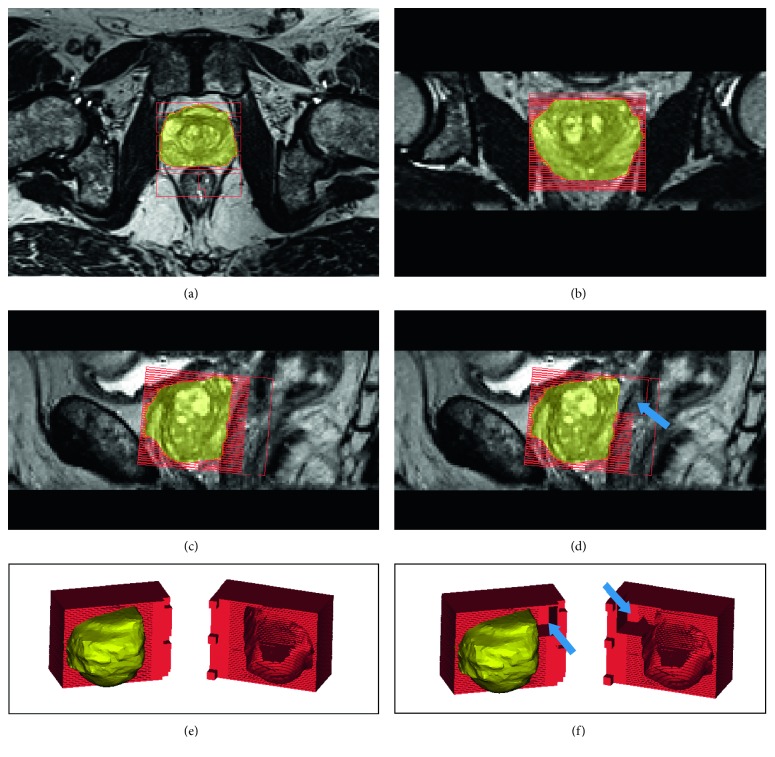
Visualization of the slicer overlaid to the MRI of the prostate imaged in three different planes and after 3D modeling. This procedure enables simple checking for correct positioning and alignment of the slicer with respect to the image acquisition axes. (a, b) Axial and coronal plane. (c) Sagittal plane with slicer without the seminal vesicles space. (d) Sagittal plane, a model that takes also into account a space (arrows) for the seminal vesicles after prostatectomy. (e, f) The 3D view of slicers with the prostate, without and with (arrows) space for seminal vesicles.

Different imaging modalities are commonly stored in a standardized Digital Imaging and COmmunications in Medicine (DICOM) format [15] that can be processed for segmentation of the organ of interest, either in a manual or in a semiautomatic manner [16]. Through dedicated computer-aided design (CAD) software, it is then possible to digitally create a cubic box, also known as a slicer, that envelops the segmented volume and provides the pathologist with customized cutting planes which match slice thickness (and possibly gaps between imaging slices) used during in vivo imaging [17] (Figure 1). By feeding the CAD file to the 3D printer software, a 3D slicer for studying imaging-histopathological correlation can be easily generated [17]. 3D printing is an additive manufacturing (AM) technology which is being increasingly applied to the biomedical field and is able to realize objects by depositing, layer by layer, plastic or metallic material as well as powders, resins, or liquids. Organ volume is then defined within the slicer by subtraction, hence creating the cavity that will receive the excised organ for histopathological assessment.

**Figure 2:**
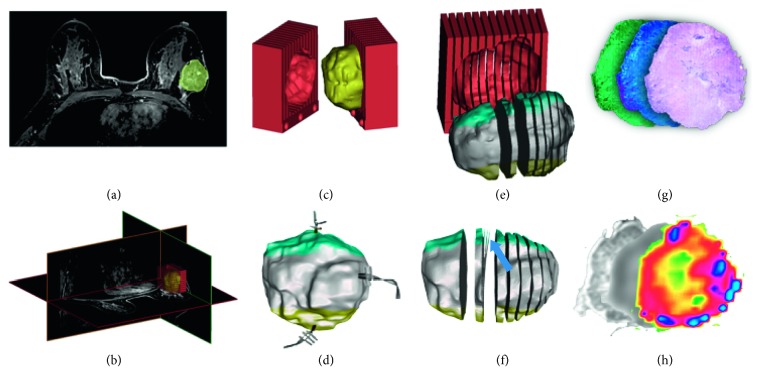
(a) Breast lesion detection and outline; (b) 3D lesion segmentation and slicer design; (c) lesion modeling and slicer prototyping; (d) lesion surgical excision with directional markers; (e) thick lesion slicing according to MR protocol; (f) thin slicing for histological assessment (blue arrow); (g) histological staining and immunohistochemistry; (h) structural, diffusion, and perfusion maps (represented in pseudocolor) corresponding to histological section.

Different 3D printing technologies exist, the main difference being the deposition method. The choice of one technology over another mainly depends on the material of choice. The most widespread technologies are: selective laser sintering (SLS), digital light processing (DLP), stereolithography (SLA), and fused deposition modeling (FDM) [18]. SLS technology uses a laser beam to polymerize materials within a closed chamber. With this technique, objects can be made from thermoplastic materials, ceramic, or silica powders. A specific SLS subcategory is represented by the DIrect Metal Printing (DMP) or Direct Metal Laser Sintering (DMLS), which allows the development of metal prototypes [18]. DLP printers use a light source (LED or LCD) to polymerize a photosensitive resin. SLA technology is different from DLP only in that it uses a UV laser [18]. Finally, FDM printers employ the heating of a plastic filament, which is deposited layer by layer through 3D movement of the printing head. This technology is the most widespread, due to ease of use and low operating cost. The most commonly used thermoplastics are acrylonitrile butadiene styrene (ABS), poly (lactic acid) (PLA), nylon, and many other materials (Figure 2) [18].

The main advantage of 3D printing is the freedom and speed of production of more or less complex objects, as well as the great accuracy of the details affordable at relatively low costs [19]. In this context, based on cost, functionality, and applications, 3D printers can be divided into consumer (desktop) and professional devices [20].

Most low-cost desktop 3D printers rely on FDM technology. They are similar to their high-end industrial counterparts as both are based on material extrusion and layer-to-layer deposition of molten thermoplastic material, and the main differences are mostly found in geometric tolerances and accuracy. Industrial printers execute calibration algorithms before each print, include a heated chamber to minimize the effects of rapid cooling of the molten plastic (e.g., warping), and can operate at higher print temperatures. Most of these machines support double extrusion. This allows the deposition of a water-soluble support material, which is removed during postprocessing; this technique results in smoother surfaces and facilitates the printing of complex parts. On the other hand, FDM 3D desktop printers are gaining more and more market share, with some high-end models supporting advanced features (e.g., calibration algorithms, heated chamber, higher print temperatures, and double extrusion). A well-calibrated basic desktop FDM machine can produce parts with fairly high spatial accuracy (typically with tolerances of ±0.5 mm) and with spatial resolution of about 0.4 mm (compared to the 0.1 mm for FDM industrial printers). Other differences are in printing speed, print area size, and cost [20]. For example, for designs that require higher spatial resolution, or for engineering materials with specific properties (thermal or chemical resistance) or large dimensions (greater than 200 mm × 200 mm × 200 mm), FDM 3D industrial printers represent the best FDM solution.

## 3. Practical Issues: The Radiological and Pathological Point of View

While histological assessment represents the gold standard for tumor characterization and prognosis, imaging techniques are emerging as valuable tools for tumor staging and follow-up, also due to their lack of (or minimal) invasivity [2, 21, 22]. Accordingly, the scientific as well as clinical communities are pushing oncological imaging to generate more specific and sensitive biomarkers of pathology or of its progression [23, 24]. For these reasons, while advanced imaging techniques for tumor proliferation, cellularity, receptor expression, or perfusion are developing and will represent a major breakthrough in clinical routine, proper validation and combination of these new parameters with the histological gold standard appears mandatory [23–25]. In this context, several points are crucial to be able to correctly compare and relate imaging to histological data.

### 3.1. The Radiological Point of View

The starting point for the mentioned workflow is the acquisition of diagnostic images and the accurate segmentation of the organ/lesion for histological analysis of the excised specimen (Figure 1) [16, 26, 27]. In this context, one key point is the choice of acquisition protocol in order to acquire volumetric images appropriate to create a faithful 3D model of the surgical piece [27]. Here, the use of isotropic voxels without interslice gap is not mandatory because the intrinsic geometry of the slicer includes several gaps to guide the pathologist during cutting and blade crossing. Should diagnostic protocols include different kind of images and modalities, the design of the cutting box will be further adapted, modifying the mold's ratio between slice thickness and gap (Table 1).

Patient position (e.g., prone position for breast acquisition) and the use of a dedicated coil (e.g., endorectal coil for the prostate investigation in MR) are also to be taken into account during the reversal engineering of the segmented volume. These factors could determine a compression/stretching effect on the volume of interest during scanning which may result in a mismatch between the in vivo/3D model and the ex vivo excised specimen [7]. In these cases, correction factors have to be defined and implemented in the model design. In this context, another possible source of bias during segmentation (mainly for the excision of lesions and less for whole organs like the prostatic gland) is the correspondence between the segmentation performed by the radiologist and the effective volume of tissue removed to be included in the cutting box. Recently, several authors compared segmented and excised volumes demonstrating that MRI constantly underestimates the extent and size of prostate cancer, with an excised tumor volume up to 3 times larger than the segmented one [36, 43, 44].

More studies aimed to increase the probability of correctly identifying a tumor focus whose existence was demonstrated histopathologically as well as to increase the specificity of imaging techniques (defined as the probability of correctly identifying the negative regions for the tumor) are currently in progress [36].

### 3.2. The Pathological Point of View

The other face of the workflow is the histopathological assessment of the excised sample. The first conceptual issue for the pathologist is to shift from thin sections, usually prepared for microscopic evaluation (about 20 *μ*m), to macrosections with the same thickness as imaged slices (generally around 2 mm) [45]. This departure from standard procedures requires changes in equipment for fixation, inclusion, and cutting of the sample, changes in staining protocols, and finally a specific training for the pathologist. The main issue regarding sample cutting is in freehand slicing. In view of the heterogeneous shape and consistency of the specimen, this practice can produce slices with different and not homogeneous thicknesses [13, 46]. Another drawback could be the presence of calcifications within the sample, which could determine deformations or even a deviation from the axis chosen for the cut.

## 4. Oncological Applications

The search was performed on both PubMed and Google Scholar, using both MeSH and free text words, including “3D printing,” “patient specific mold,” “cutting boxes,” “histopathologic correlation,” and “3D printed molds” terms. Further studies were identified though citations within articles found and using the PubMed “related citations” function. This search resulted in 20 articles: 14 dealt with the prostate, 4 with the brain (of which 2 in a preclinical setting), 1 with the liver, and 1 with the kidney. The distribution of type of printer used in these papers is similar to the distribution of the organs involved. FDM is the dominant technology, probably due to its low cost and ease of management and use. Printing times vary from organ to organ. For the prostate, the printing time varies from 5 to 24 h, with much longer times for the human brain (about 70–100 h), the liver (45–72 h), and, in contrast, 3–12 h for the marmoset brain.

### 4.1. Prostate Cancer

The main application of customized slicers in biomedicine is in prostate cancer pathology, also in view of the clearly visible margins of the gland compared to the surrounding tissues that facilitate a reliable segmentation before prostatectomy [35, 37–41] (Figures 1 and 2). Previous studies have attempted to define various orientation and sectioning techniques to coregister histological sample images to MR images [47]. Villers et al. employed anatomical landmarks like gland contours [48]. Other authors employed wider inclusion criteria, identifying the overlap between imaging and histology with a tolerance of 3–10 mm [49]. However, none of these strategies was tailored to the in vivo prostate shape assessed by MRI nor considered gland deformation during cutting [50, 51]. The first paper which employs a 3D printer for these purposes dates back to 2009 [11]. In this paper, the authors highlight the difficulty in dissecting the prostate in concordance with MRI once that the anatomical orientation of the body is lost, stressing the need of short times between imaging acquisition/mold design and prostatectomy due to the time-dependent variability in size in benign prostatic hyperplasia [52, 53].

Bourne et al. implemented a strategy that overcomes uncertainties about size variability by creating two versions of the mold which include *a* + 10 and *a* − 10% variation of the volume along the axial plane, respectively [7]. Elen at al. [34] suggested the positioning of a urethral catheter in the sample/box, in order to reduce the likelihood of misalignment due to rotation offset, at the cost introducing a small distortion of the tissue around the catheter. In the same article, the authors introduced a second, high-resolution ex vivo acquisitions of the sample within the box before histological assessment [34]. Using the same dual-point acquisition, Priester et al. [33] found a 16% volumetric reduction of the ex vivo MRI compared to the contouring performed on the in vivo MRI, with almost 80% of spatial overlap. The problem of volumetric reduction, in addition to the shrinkage due to formalin fixation, appeared to be determined by the practice of surgical resection. In a previous study on 114 patients, the same authors showed a constant underestimation of the extent and size of prostate tumors in MRI, and these results were confirmed by other authors who found differences of up to 150% between imaging and histological tumor volume [36, 43, 44] but also contrasted by other ones [28, 54] (Table 1).

Other studies, thanks to the use of the slicer, were able to expand the integration of data between histology and radiology, a new “radiopathomic” approach to map prostate cancer. The digitization of pathology results with automatic acquisition of the histological sample, combined with MRI, allowed the authors to build predictive maps of the histological features. This “radiopathomic mapping” technique might be also relevant for dose-painting strategies in prostate radiotherapy [55]. In another study [56], the authors demonstrated a method to correlate histopathology to in vivo PET/MRI in prostate cancer, coregistering the Gleason score maps to MRI sequences and PSMA PET images [56].

### 4.2. Brain

Imaging-histological correlation in the brain represents a promising goal for the understanding of neurological diseases pathogenesis, mainly in view of the invasiveness of histological sampling through biopsy or the late postmortem assessment [57]. Moreover, shape of the brain makes the cutting phase extremely complex, even just in view of the difficulty of keeping the sample flat on a surface.

Previous attempts to address this issue were based on the use of deformation algorithms [58] or placement of fiducial markers [29]. Absinta et al. [59] employed 3D printing technology to relate standard in vivo imaging to histopathological sections through an intermediate step represented by a postmortem MRI scan. This approach was chosen to limit distortion due to movement during the cutting phase and formalin fixation which, as mentioned above, induces a sample shrinking which can range from 15% to 30% [60].

**Figure 3:**
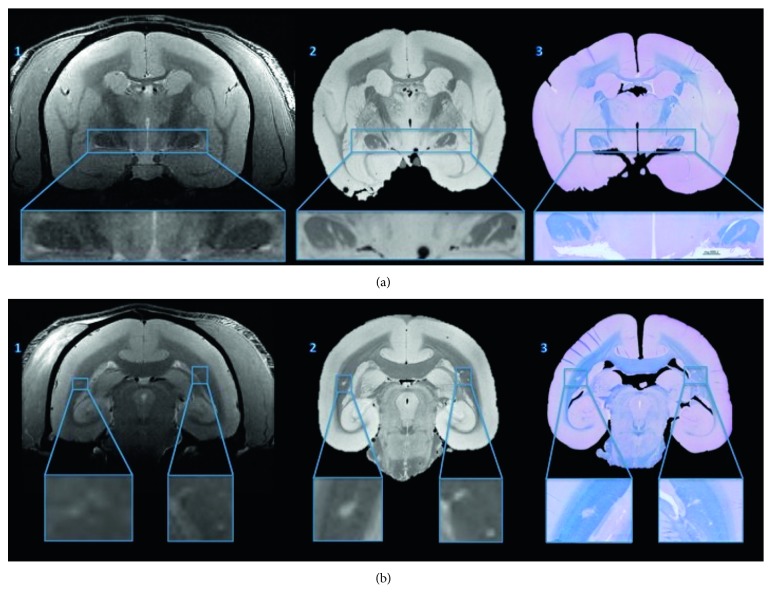
From left to right: in vivo MRI (1), postmortem MRI (2), and fast blue/cresyl violet histological staining for myelination assessment (3) at two different coronal planes (a, b) (figure adapted from [32] under Creative Commons (CC) 3.0 license).

Other studies have attempted to employ this technology with the same goals also in preclinical settings [31, 32, 61] (Figure 3).

### 4.3. Other Applications: Liver and Kidney

Trout et al. [30] employed a similar reverse engineering and 3D printing workflow to associate imaging and histology in 13 patients who underwent hepatectomy (10 subsequently underwent transplantation, 3 partial hepatectomy). The authors concluded that, for the inclusion of an organ piece and not of the whole organ, the lack of orientation references for the pathologist is a “tricky” issue and that the use of a customized slicer can be of significant aid [30]. Similarly, Dwivedi et al. [42] applied different design methods along with 3D printing to the study of renal masses in 6 patients. For the first patient, 2 different molds were designed, one with the outer contour of the tumor alone and the other with the surrounding parenchyma. After this first attempt, the authors decided to create (for the next 5 patients) a single box, based only on the external contour of the tumor. In contrast to studies in other regions, the authors reported a more complex imaging-histological correlation for tumors with a cystic component, due to fluid loss or collapse during the cutting phase [42].

## 5. Future Perspectives

3D printing technology applied to the biomedical field has the potential to both facilitate the pathologist in the cutting phase and to enable associational studies between imaging and histological data. The opportunity to relate macroscopic information to microscopic tissue properties through the establishment of spatial correspondences can be crucial both to validate existing imaging techniques and to identify new promising biomarkers for diagnosis, treatment target identification, and prognosis. Moreover, the finding of a significant underestimation of oncological lesions in the case of imaging [38] reappraises the increasing role of radiology in the clinical workflow [38], providing new avenues for the discovery of quantitative biomarkers and for a better understanding of lesion recurrence after treatment.

In summary, reverse engineering by imaging scans and 3D printing of CAD models will make a significant contribution to improving both the reliability of imaging modalities and the quality of histological assessment in cancer detection.

## Figures and Tables

**Table 1:** Review of literature employing personalized 3D printed molds for imaging-histological correlation.

Article	Organ	Patients	MR imaging parameters slice/gap (mm)	Slicer design (mm)	Printer	Pro/desktop	Price	Printing technology	Layer resolution (microns)	Material	Printing time (h)
[28]	Brain	4	N/A	N/A	MakerBot 5th Generation Replicator	Desktop	$2,500	FDM	100	PLA	N/A
[29]	Brain	3	0.6/0	4.8/1.2	Fortus 360mc	Professional	$52,000	FDM	127	ABS	100
[30]	Liver	13	5/0	10/2	Replicator Z18	Desktop	$6,500	FDM	100	PLA	45–72
[31]	Marmoset brain	5	0.6/0 in vivo–0.15/0 ex vivo	2.5/0 + 1 slice 5/0	ProJet 3510	Professional	$70,000.	MJP	127	VisiJet	8
[32]	Marmoset brain-human brain	N/A	0.1/0 marmoset–0.3/0 human	3/0.5 marmoset–6/1.2 human	Ultimaker 2	Desktop	$2,500	FDM	20	PLA	3–12 marmoset–70 human
[17]	Prostate	25	N/A	5/0	ProJet 3510 HD Plus-Leapfrog Creatr XL	Professional-desktop	$70,000–$6,000	MJP-FDM	16–50	VisiJet-PLA	N/A
[33]	Prostate	10	4.5/0	1.5/0 phantom–1.5/0 ex vivo	MakerBot	Desktop	$2,500	FDM	200	PLA	5
[34]	Prostate	6	3/0 in vivo–0.18/0 ex vivo	3/0	UP3DUP Plus	Desktop	$1,100	FDM	150	N/A	N/A
[35]	Prostate	1	N/A	4.5/0.5	MakerBot Replicator 2	Desktop	$1,600	FDM	100	PLA	6
[36]	Prostate	114	1.5/0	4.5/0.5	MakerBot Replicator 2	Desktop	$1,600	FDM	100	PLA	6
[37]	Prostate	8	3/0	6/0	N/A	N/A	N/A	N/A	N/A	N/A	N/A
[38]	Prostate	31	3/0 ex vivo–6/0 in vivo	6/0 and 3/0	N/A	N/A	N/A	N/A	N/A	N/A	N/A
[7]	Prostate	6	2.5/0 or 2.5/2.5 mm in vivo–1/0 ex vivo	0.4/5	EOSINT P100	Professional	$175,000	SLS	60	PA2200	N/A
[9]	Prostate	N/A	3/0	6/1	Dimension Elite 3D printer	Professional	$31,900.00	FDM	178	ABS	8 to 24
[39]	Prostate	1	3/0	6/1	Dimension Elite 3D printer	Professional	$31,900.00	FDM	178	ABS	8 to 24
[36]	Prostate	45	3/0	6/0	Dimension Elite 3D printer	Professional	$31,900.00	FDM	178	ABS	8 to 24
[40]	Prostate	26	3/0	6/0	Dimension Elite 3D printer	Professional	$31,900.00	FDM	178	ABS	8 to 24
[41]	Prostate	40	N/A	N/A	Dimension Elite 3D printer	Professional	$31,900.00	FDM	178	ABS	N/A
[39]	Prostate	1	N/A	6/1	Dimension Elite 3D printer	Professional	$31,900.00	FDM	178	ABS	8
[42]	Renal masses	6	N/A	4-5/0	ProJet 3512HD	Professional	$70,000	MJP	127	VisiJet	12–14
